# Health behaviour change during the UK COVID‐19 lockdown: Findings from the first wave of the C‐19 health behaviour and well‐being daily tracker study

**DOI:** 10.1111/bjhp.12500

**Published:** 2021-01-06

**Authors:** Felix Naughton, Emma Ward, Mizanur Khondoker, Pippa Belderson, Anne Marie Minihane, Jack Dainty, Sarah Hanson, Richard Holland, Tracey Brown, Caitlin Notley

**Affiliations:** ^1^ Behavioural and Implementation Science Group School of Health Sciences University of East Anglia UK; ^2^ Addictions Research Group Norwich Medical School University of East Anglia UK; ^3^ Norwich Medical School University of East Anglia Norwich UK; ^4^ Nutrition and Preventive Medicine Group Norwich Medical School University of East Anglia UK; ^5^ Leicester Medical School University of Leicester UK

**Keywords:** COVID‐19, health behaviours, behaviour change, intensive longitudinal design, ecological momentary assessment

## Abstract

**Objectives:**

To provide baseline cohort descriptives and assess change in health behaviours since the UK COVID‐19 lockdown.

**Design:**

A prospective cohort (*N* = 1,044) of people recruited online, purposively targeting vulnerable populations.

**Methods:**

After a baseline survey (April 2020), participants completed 3 months of daily ecological momentary assessments (EMA). Dietary, physical activity, alcohol, smoking, vaping and substance use behaviours collected retrospectively for the pre‐COVID‐19 period were compared with daily EMA surveys over the first 30 days during early lockdown. Predictors of behaviour change were assessed using multivariable regression models.

**Results:**

30% of the cohort had a COVID‐19 at risk health condition, 37% were classed as deprived and 6% self‐reported a mental health condition. Relative to pre‐pandemic levels, participants ate almost one portion of fruit and vegetables less per day (vegetables mean difference −0.33, 95% CI −0.40, −0.25; fruit −0.57, 95% CI −0.64, −0.50), but showed no change in high sugar portions per day (−0.03, 95% CI −0.12, 0.06). Participants spent half a day less per week doing ≥30 min of moderate to vigorous physical activity (−0.57, 95% CI −0.73, −0.40) but slightly increased days of strength training (0.21, 95% CI 0.09, 0.34), increased alcohol intake (AUDIT‐C score change 0.25, 95% CI 0.13, 0.37), though did not change smoking, vaping or substance use behaviour. Worsening health behaviour change was associated with being younger, female and higher body mass index.

**Conclusions:**

The cohort reported worsening health behaviours during early lockdown. Longer term changes will be investigated using further waves of data collection.

**Statement of contribution**

***What is already known on this subject?***

1. Drinking, smoking, diet, and physical activity behaviours are collectively responsible for almost 50% of all‐cause years of life lost in the United Kingdom.
2. Within‐ and between‐individual changes in health behaviours are essential to track as they critically impact long‐term health and well‐being.
3. Changes in health behaviours due to the COVID‐19 lockdown across multiple countries have been reported.

***What does this study add?***

1. This is the first UK study using ecological momentary assessment (EMA) to track health behaviours during the COVID‐19 lockdown.
2. The cohort ate less fruit and vegetables, reduced their cardiovascular activity but increased strength training during early lockdown.
3. Alcohol consumption increased but we found no differences in smoking, vaping, or substance use.
4. Worsening health behaviours were associated with being younger, female, and having a high BMI.

## Background

Health behaviours such as alcohol (Griswold et al., 2018), tobacco and substance use (NHS, 2018), dietary choices (Steel et al., 2018), and physical activity (Reiner, Niermann, Jekauc, & Woll, 2013) have direct impacts on immediate and long‐term health outcomes and together account for almost half of all‐cause years of life lost in the United Kingdom (Murray et al., 2013). Health behaviours also directly affect mental health and risk of chronic conditions, including cancer, cardiovascular disease, and type II diabetes. In addition to being potentially important determinants of contracting and surviving viral infections such as COVID‐19, and severity of symptoms, much of the severe pathogenesis of COVID‐19 disease progression is now known to be linked to a hyperinflammatory immune response (‘cytokine storm’), which may be negatively impacted by poorer patterns of health behaviours (Sinha, Matthay, & Calfee, 2020).

The initial 'lockdown' measures imposed in the United Kingdom and many countries worldwide swiftly enforced significant changes and constraints on daily living, such as social distancing and limits on people leaving their homes, that are very likely to have impacted profoundly on enacted health behaviours. There are already indications worldwide that social measures have significantly impacted health behaviours such as dietary choices (Deschasaux‐Tanguy et al., 2020) and well‐being (Lades, Laffan, Daly, & Delaney, 2020). However, the full and long‐term impact of the unprecedented social measures in the United Kingdom is unknown. Moreover, there has been less focus on health behaviours compared with mental health (Arora & Grey, 2020).

The United Kingdom entered the COVID‐19 pandemic in March 2020 and the Government announced social distancing and then full lockdown measures across the population on 23^rd^ March, which came into force on 26^th^ March (the full regulation is at https://www.legislation.gov.uk/uksi/2020/350/regulation/1/made). In the following weeks, initial reports of changes to health behaviour began to emerge. Sales figures suggested a sharp increase in alcohol consumption (Inman, 2020), but simultaneously sales of alcohol through social venues such as pubs and clubs stopped completely as venues closed. This suggests that both absolute alcohol consumption and patterns of alcohol use may have changed. Even relatively small changes in alcohol consumption can have a marked impact on long‐term health (Mostofsky, Mukamal, Giovannucci, Stampfer, & Rimm, 2016).

Similarly, sales of tobacco increased by 9% in the United Kingdom during March 2020 (Evans, Middlehurst, & Nilsson, 2020), but factors prompting individual cessation attempts are complex. On the one hand, reports of emerging evidence linking poor COVID‐19 outcomes and smoking (van Zyl‐Smit, Richards, & Leone, 2020), and the UK ‘Quit for COVID’ campaign (Smokefree Action Coalition, 2020a) may have precipitated increased smoking quit attempts (Klemperer, West, Peasley‐Miklus, & Villanti, 2020; Smokefree Action Coalition, 2020b). On the other hand, stress may negatively impact motivation to quit or indeed may be a precursor for relapse to tobacco smoking for recent ex‐smokers (Kim et al., 2019). In addition, there have also been reports of a potential protective effect of nicotine from COVID‐19 complications (Farsalinos et al., 2020), which were reported by some media as possible indication of a protective effect from smoking. As tobacco smoking remains the leading cause of preventable death (World Health Organization, 2018), any change in cessation attempts or increase in smoking relapse will have a direct impact on long‐term morbidity and mortality (Pirie, Peto, Reeves, Green, & Beral, 2013). Vaping is promoted in the United Kingdom as a safer alternative to smoking (Public Health England, 2020a), and, although the evidence on any potential impact of vaping on COVID‐19 risks is unknown, advice has remained unchanged (Public Health England, 2020b).

Initial evidence suggests that food purchasing, cooking, and dietary behaviour during lockdown may have been markedly impacted (FSA, 2020; Venema, 2020), with people tending to choose high sugar calorie‐rich foods as a response to stress, or eating/snacking more as a result of changed work/life patterns, such as working from home (Defra, 2020; Institute for Employment Studies, 2020). A well‐balanced, nutrient‐rich diet is an important mediator of immune function, and a well‐functioning immune system is a key determinant of COVID‐19 prognosis if infected (Calder, 2020). Furthermore, excess body weight is associated with higher infection rates and a more severe COVID‐19 prognosis (Anderson et al., 2020; Razieh, Zaccardi, Davies, Khunti, & Yates, 2020). This is significant as two‐thirds of the UK population are overweight or obese, with half self‐reporting weight gain during lockdown (Duffy, 2020). Weight, and so risk from COVID‐19, is also affected by physical activity. Initial evidence indicates a varied influence of lockdown on activity, with activity levels varying based on characteristics such as age, education and prior pre‐pandemic activity levels (Constandt et al., 2020) and reports of higher reductions in activity among lower‐income US adults (Dunton, Wang, Do, & Courtney, 2020). Increases in sedentary time have also been reported, in part through being constrained by the home environment (Deschasaux‐Tanguy et al., 2020), with worse weight related behaviours associated with lower educational qualifications and income.

Negative changes in health behaviours are likely to interact in complex ways. For example, those smoking tobacco may also be likely to drink more alcohol and engage in less physical activity (Noble, Paul, Turon, & Oldmeadow, 2015). Additionally, these interacting behaviours are very likely to be associated with existing multiple disadvantage and health inequalities, such as lower socioeconomic status (Abrams & Szefler, 2020; Health Foundation, 2020). It is imperative that we track and understand changes in health behaviours over time, and particularly how these changes may disproportionately impact more vulnerable groups in order to ultimately estimate the likely impact on long‐term health.

Emerging evidence, to date, provides a limited picture on COVID‐19 pandemic‐related health behaviour change in the United Kingdom. This is because of a reliance on proxy measures of behaviour, such as product sales, cross‐sectional studies, and a limited number of UK studies. To our knowledge, there are as yet no daily tracking studies monitoring health behaviour change over time during the unique timepoint encompassing social distancing and lockdown. Daily tracking enables assessment of the impact of government mandated social distancing (Arora & Grey, 2020) while minimizing recall bias inherent in self‐reported behavioural measurement. In this first article, we identify initial changes in health behaviours due to lockdown, relative to pre‐pandemic levels. This will establish a cohort ‘baseline’ in early lockdown, which is crucial for identifying longer term changes in behaviour and subsequent impacts on health and mental health. We have aggregated the daily tracking data at the individual level in order to provide this early lockdown baseline as a comparator for follow‐up measurements and in order to provide an estimate of behaviour change relative to pre‐lockdown levels. We also examine whether changes in health behaviours in early lockdown differ between different groups, particularly focusing on vulnerable populations experiencing health disparity (Public Health England, 2020c). This understanding would help inform policy and practice changes that may be applicable to future pandemics, but also aid preparation for mitigating any potential negative impacts of short‐term health behaviour change observed.

## Methods

### Design and participants

This is a prospective cohort study of UK residents recruited online in early April 2020. This paper includes baseline data and daily surveys using ecological momentary assessment (EMA) collected for the first 30 days out of the total of 12 weeks of EMA data collection. These ‘first wave’ findings use the first 30 days of EMA data only as we wanted to assess behaviours during early lockdown.

We purposively targeted vulnerable populations for recruitment and focus on three priority groups in this study: those with a physical high‐risk health condition for COVID‐19 (in line with the UK National Health Service definitions), those living in a high deprivation area, and those with a self‐reported mental health issue.

Participants, who were 18 years or older, lived in the United Kingdom, and had access to a smartphone, were recruited online, using social media and targeted using key contacts as gatekeepers to vulnerable groups (e.g., women’s shelters, mental health support groups). No financial incentives were provided for participation, although a summary report of each participant’s daily surveys was offered. The cohort was recruited over 12 days (8^th^ April to 19^th^ April 2020), and the 30‐day daily survey period spanned 10^th^ April to 18^th^ May. Participants responded to study adverts by clicking a weblink, were fully informed about the purpose of the research, and gave consent to take part at the beginning of the baseline survey. All surveys were online, hosted on Qualtrics XM software. One to two days after participants completed the baseline questionnaire, daily text reminders were initiated for 12 weeks using an automated SMS system for completion of daily health behaviour monitoring at either 8pm, 9pm, or 10pm, depending on preference. The SMS messages asked participants to submit their survey for behaviours each day before bedtime, and surveys submitted the next day prior to the next SMS were included. A small number of participants requested survey links to be sent by email instead. Ethical approval was granted by the Faculty of Medicine and Health research ethics committee on the 31^st^ March 2020 (2019/20‐089).

### Measurement

Baseline: All participants gave comprehensive demographic information, domestic circumstances, employment and income information, including keyworker status, self‐reported pre‐existing health conditions including pregnancy, coronavirus at‐risk status, either ‘extremely vulnerable’ or ‘vulnerable’ according to NHS definitions (Public Health England, 2020d, 2020e), reports of COVID‐19 relevant symptoms (continuous cough and/or fever as it was at the time of measurement) and diagnosis, any self‐reported mental health issues and smoking status. We used data asked at baseline on the following before the COVID‐19 pandemic: typical portions of vegetables, fruits and high sugar foods per day based on Public Health England portion size definitions, self‐rated diet quality (on a five‐point scale from poor [1] to excellent [5]) (Adjoian, Firestone, Eisenhower, & Yi, 2016; Loftfield, Yi, Immerwahr, & Eisenhower, 2015), number of days per week undertaking at least 30 minutes of moderate to vigorous physical activity (MVPA) (Milton, Clemes, & Bull, 2013) and strength training, the World Health Organization version of the Alcohol Use Disorders Identification Test for Consumption (AUDIT‐C) (Bradley et al., 2007), smoking status and typical number of cigarettes smoked per day (Heatherton, Kozlowski, Frecker, & Fagerström, 1991) and frequency of vaping and substance use (split into cannabis, stimulants, depressants, hallucinogens and new psychoactive substances), based on AUDIT‐C frequency categories. For vaping and substance use, we asked participants to report any use ‘within the 3 months preceding the COVID‐19 pandemic (Nov 2019 to Jan 2020)’. We collected weight and height in order to calculate BMI.

Daily surveys: We used data on reports of how many portions of vegetables, fruits and high sugar foods participants consumed and self‐rated diet quality each day (in line with baseline measures) for the first seven daily surveys. This was then aggregated into a mean value per person per day. Similarly we used daily reports of minutes of MVPA and strength training over the first seven daily surveys to calculate the number of days where 30 minutes or more of MVPA and strength training was undertaken. We used daily reports, over 30 days, on the number of alcoholic drinks consumed each day, including any drinks planned for later that evening. These data were then used to calculate the equivalent AUDIT‐C score over the 30‐day period for each participant, including two of the three most relevant component categories; number of drinks consumed on a typical days drinking and frequency of days where alcohol is consumed. Any smoking and the number of cigarettes smoked over 30 days of daily surveys was used to determine smoking and relapse behaviour, respectively, as is common in EMA studies (Shiffman, 2009). Data collected on any vaping or substance use each day were used to determine vaping and substance use behaviours (Shiffman, 2009).

The full surveys are available on the Open Science Framework (https://osf.io/dm853/).

### Analysis

Descriptive statistics were generated for participant characteristics. Pre‐COVID‐19 pandemic mean values and proportions and lockdown mean values and proportions (‘post‐COVID‐19’) were calculated along with the mean/proportion difference with 95% confidence intervals. This was calculated for the whole sample and then separately for the three priority groups for all health behaviour measures. Multivariable regression models (either linear, logistic, negative binomial or Poisson regression as appropriate) were then used with each of the health behaviour variables post‐COVID‐19 as dependent variables, and the pre‐COVID‐19 variables for the corresponding behaviour as a covariate. Additional covariates determined a priori were simultaneously included in the models and included having a COVID‐19 at‐risk health condition, index of multiple deprivation (IMD) decile, self‐reported mental health issue, keyworker status, childcare responsibility during work hours, living alone, age, gender (male vs. female), BMI and ethnicity (White vs. other). This follows our published statistical analysis plan (https://osf.io/9yfu8/), with the exception that we added ethnicity as an additional covariate.

#### Missing data

For dietary behaviour and the number of cigarettes smoked per day, we calculated the mean values while ignoring any missing days over the 7 day period used, which was deemed sufficient for sampling and estimating these behaviours. For the behaviours that would not necessarily occur daily – physical activity, drinking, smoking (binary), vaping, and substance use – we applied a conservative missing equals not having undertaken the behaviour rule.

We encountered missing data in outcome variables, but not in the (baseline) covariates, and imputation is not typically the method of choice for dealing with missing data in outcomes (Sullivan, White, Salter, Ryan, & Lee, 2018). We identified two potential drivers (predictors) of missingness in our data (gender and smoking status predicted survey completion over 7 days and smoking status over 30 days), which (as is common in diary studies) indicated a ‘missing at random’ (MAR) assumption to be the most plausible mechanism of missingness. We then followed recommendations from Groenwold, Donders, Roes, Harrell, and Moons (2012) to include predictors of missing data as covariates in regression models to eliminate any potential bias caused by missing data.

Further, as a sensitivity analysis, we followed recommendations by Bolger and Laurenceau (2013) to compare the findings with varying levels of missing data, to assess the impact of MAR assumption and our handling of missing data. We re‐ran the analyses of changes in behaviour pre and post‐pandemic under two missing data scenarios. For the first scenario, we included only participants who had more than 50% of completed data; 4 or more surveys over 7 days and 15 or more surveys over 30 days for the respective measures. We then ran a more extreme scenario including only participants with 7 surveys over 7 days and 25 surveys over 30 days.

## Findings

### Sample summary

Out of 1,697 visits to the baseline survey webpage, 1,044 (62%) people participated and completed the baseline survey. Table 1 provides a summary of the participants’ baseline characteristics (Table 1). The majority were female (73%), White (96%), and lived in England (97%). Looking at the three key subgroups focused on, 30% had a COVID‐19 at‐risk health condition, 38% were from higher deprivation neighbourhoods, and 6% had a self‐reported mental health issue. Of those completing the baseline questionnaire, 95% (987) completed at least one daily survey with a median completion rate among these of 28 out of 30 (93%) surveys. In total, 72 (6.9%) of participants withdrew, although some completed daily surveys.

**Table 1:** Sample description (*N *= 1,044)

Characteristic	%^a^	*N* ^b^
Age category (years)		
18–24	10.9	113
25–44	36.3	377
45–64	41.1	427
65+	11.8	123
Gender		
Male	27.1	279
Female	72.7	747
Other	0.2	2
Ethnicity		
White	95.7	996
Other	4.3	45
Country of residence		
England	96.9	998
Jersey	0.1	1
Northern Ireland	0.4	4
Scotland	1.7	17
Wales	1.0	10
Number of adults in household		
1 (living alone)	21.2	221
2	56.6	590
3	13.8	144
4+	8.3	87
Children in household		
Yes	31.3	327
No	68.7	717
Childcare responsibilities during worktime		
Yes	18.1	189
No/no children	81.9	855
Keyworker		
Yes	26.6	278
No/not working	73.4	766
Employment status		
Employed/self‐employed/freelance	60.0	626
Not working (student/home carer/retired)	22.9	239
Never worked or long‐term unemployed	0.2	2
Unemployed and looking for work (not due to COVID‐19)	2.1	22
Out of work/furloughed/leave of absence (due to COVID‐19)	11.8	123
Unable to work because of sickness or disability	3.1	32
Income change (from employment) since pandemic		
Decreased	9.5	99
Stayed the same	48.1	502
Increased	2.4	25
Not employed	40.0	418
Household income lower than living wage (£1,500 net per month)^c^		
Yes	21.6	198
No	78.4	718
Index of Multiple Deprivation (IMD) quintile (1 = most deprived)^d^		
1	11.7	120
2	15.0	154
3	24.7	253
4	22.9	234
5	25.7	263
COVID‐19 at‐risk health condition		
Very high‐risk health condition	6.7	70
Increased risk health condition	22.8	238
No increased risk health condition	70.5	736
Received COVID‐19‐positive diagnosis		
Yes	0.2	2
No	99.8	1042
Experienced COVID‐19 symptoms since pandemic began (Feb 2020)		
Yes	16.7	174
No	83.3	870
Self‐reported mental health issue		
Yes	6.0	63
No	94.0	981
Obese (BMI 30kg/m^2^ or greater)		
Yes	25.4	262
No	74.6	770
Smoking status		
Current smoker	8.6	90
Quit smoking since pandemic started	1.0	10
Quit smoking before pandemic started	26.7	278
Non‐smokers	63.8	665

Note

^a^ Percentages may not add up to 100 due to rounding.

^b^ Due to missing data, totals may not add to 1,044.

^c^ Large amount of missing data (*n *= 916).

^d^ Combined using IMD decile scores from England (2019), Northern Ireland (2017), Scotland (2020), and Wales (2019).

### Changes in dietary behaviour

Across the sample as a whole and relative to pre‐COVID‐19 portions, there were reductions in the mean reported number of daily portions of vegetables (mean difference −.33, 95% CI −0.40, −0.25) and fruit (mean difference −.57, 95% CI −0.64, −0.50) but no change in reported portions of high sugar foods consumed (mean difference −.03, 95% CI −0.12, 0.06) during early lockdown (Table 2 ). The mean self‐rated diet quality was 15% lower during lockdown relative to pre‐COVID‐19 levels (mean difference −.45, 95% CI −0.51, −0.40). These dietary behaviour patterns were similar across the three key subgroups. Removing those who completed fewer than 50% of daily surveys over the 7 days (3 or fewer days) (Table S1) or fewer than 100% (all 7 days) made no meaningful difference to these findings.

**Table 2:** Health behaviours pre‐COVID and in the first week of daily surveys during the UK COVID pandemic for all participants and for key subgroups

Behaviour	C19 at‐risk health condition (*N *= 308)^a^			High deprivation (IMD deciles 1–5; *N *= 388)^a^			Mental health issue (*N *= 63)^a^			All participants (*N *= 1,044)^a^		
	Pre^b^	Post^c^	Difference (95% CI)	Pre^b^	Post^c^	Difference (95% CI)	Pre^b^	Post^c^	Difference (95% CI)	Pre^b^	Post^c^	Difference (95% CI)
Diet and nutrition												
Vegetable portions per day	3.3	2.9	−0.38 (−0.52, −0.24)	3.1	2.7	−0.38 (−0.50, −0.25)	2.6	2.4	−0.13 (−0.40, 0.15)	3.1	2.8	−0.33 (−0.40, −0.25)
Fruit portions per day	2.6	2.0	−0.54 (−0.68, −0.40)	2.4	1.9	−0.50 (−0.61, −0.38)	2.2	1.6	−0.58 (−0.85, −0.32)	2.4	1.9	−0.57 (−0.64, −0.50)
High sugar portions per day	2.0	1.9	−0.05 (−0.24, 0.13)	2.2	2.1	−0.10 (−0.26, 0.06)	2.2	2.1	−0.11 (−0.53, 0.30)	2.1	2.1	−0.03 (−0.12, 0.06)
Self‐rated diet quality	3.2	2.8	−0.40 (−0.51, −0.29)	3.0	2.6	−0.37 (−0.46, −0.29)	2.6	2.3	−0.28 (−0.53, −0.04)	3.1	2.7	−0.45 (−0.51, −0.40)
Physical activity												
Days of ≥ 30 mins MVPA per week	3.4	2.5	−0.91 (−1.22, −0.60)	3.3	2.4	−0.83 (−1.09, −0.57)	2.6	1.9	−0.55 (−1.18, 0.78)	3.3	2.7	−0.57 (−0.73, −0.40)
Days of strength training per week	1.4	1.4	0.08 (−0.16, 0.32)	1.3	1.5	0.26 (0.06, 0.47)	0.9	1.2	0.40 (−0.04, 0.84)	1.3	1.5	0.21 (0.09, 0.34)
Alcohol consumption												
AUDIT‐C score	2.7	3.0	0.29 (0.08, 0.50)	2.8	3.0	0.25 (0.05, 0.45)	2.3	2.6	0.34 (−0.13, 0.81)	2.8	3.1	0.25 (0.13, 0.37)
Drinks (category) consumed on a typical days drinking^d^	1.4	1.4	0.01 (−0.08, 0.10)	1.4	1.4	0.00 (−0.09, 0.09)	1.3	1.4	0.07 (−0.21, 0.35)	1.4	1.4	−0.03 (−0.09, 0.02)
Days (category) alcohol consumed per month^e^	3.0	3.3	0.26 (0.14, 0.39)	3.0	3.3	0.23 (0.12, 0.34)	2.6	3.0	0.36 (0.11, 0.61)	3.1	3.4	0.27 (0.21, 0.34)
Smoking and vaping												
Reported smoking	8.8%	9.7%	0.9% (−3.8%, 5.7%)	11.9%	13.5%	1.6% (−3.2%, 6.4%)	15.9%	20.3%	4.5% (−9.3%, 18.3%)	9.6%	10.8%	1.3% (−1.4%, 3.9%)
Cigarettes per day (among smokers)	9.3	9.0	0.6^f^ (−1.20, 2.42)	10.9	11.0	0.75^f^ (−0.76, 2.26)	14.1	14.2	0.20^f^ (−3.18, 3.57)	9.3	9.1	−0.00^f^ (−0.96, 0.96)
Any e‐cigarette use	5.5%	5.5%	0.0% (−3.8%, 3.8%)	5.9%	5.4%	0.5% (3.9%, 2.8%)	4.8%	1.6%	−3.2% (−11.6%, 4.4%)	5.3%	4.3%	−1.0% (−2.8%, 0.9%)
Substance use												
Monthly substance use	3.9%	3.6%	−0.3% (−3.5%, 2.9%)	5.4%	5.7%	0.3% (−3.1%, 3.6%)	6.4%	1.6%	−4.8% (−13.7%, 3.1%)	4.0%	3.1%	−1.0% (−2.6%, 0.7%)

Note

^a^ Denominators vary for the different behaviours due to missing data.

^b^ Self‐reported average portions per day/diet quality before COVID‐19 pandemic, collected at baseline (early April 2020).

^c^ Mean self‐reported portions consumed per day/diet quality collected daily over the first seven daily surveys after baseline (April 2020).

^d^ Only includes data from those participants reporting alcohol consumption (‘pre’ [baseline] *n *= 879, ‘post’ *n *= 794). AUDIT‐C drinking frequency categories: 1 = 1–2 drinks, 2 = 3–4 drinks, 3 = 5–6 drinks, 4 = 7–9 drinks, and 5 = 10+ drinks.

^e^ AUDIT‐C days drinking categories: 1 = never, 2 = once a month, 3 = 2–4 times per month, 4 = 2–3 times per week, and 5 = 4+ times per week.

^f^ Calculated only for those reporting smoking both at baseline and in first 7 days of daily surveys.

**Table 3:** Predictors of health behaviour change during the pandemic relative to pre‐COVID behaviour

Outcome^b^	C‐19 at risk	IMD decile^a^	Mental health issue	Keyworker	Childcare during work	Living alone	Age	Gender (male vs. female)	BMI	Ethnicity (White vs. other)
	*B* ^c^ (*p* value)	*B* ^c^ (*p* value)	B^c^ (*p* value)	*B* ^c^ (*p* value)	*B* ^c^ (*p* value)	*B* ^c^ (*p* value)	*B* ^c^ (*p* value)	*B* ^c^ (*p* value)	*B* ^c^ (*p* value)	*B* ^c^ (*p* value)
Diet and nutrition										
Vegetable portions per day	−.03 (.668)	.01 (.403)	−.03 (.816)	−.09 (.244)	.04 (.678)	−.09 (.324)	**.01 (<.001)**	.1 (.159)	−**.01 (.014)**	.12 (.479)
Fruit portions per day	−.01 (.935)	−.01 (.378)	−.02 (.906)	−.01 (.856)	−.08 (.311)	−.03 (.727)	**.02 (<.001)**	−.07 (.295)	−**.01 (.040)**	**.33 (.027)**
High sugar portions per day	−.09 (.294)	.01 (.539)	−.15 (.330)	.09 (.287)	.15 (.120)	.09 (.369)	−**.01 (<.001)**	**.18 (.030)**	**.02 (.003)**	−.21 (.255)
Self‐rated diet quality	−.04 (.456)	−.00 (.899)	−.08 (.416)	−.06 (.231)	−.00 (.989)	−.11 (.068)	**.01 (<.001)**	−**.21 (<.001)**	−**.01 (.045)**	.16 (.137)
Physical activity										
Days of ≥ 30 mins MVPA per week	−**.19 (.039)**	**.03 (.028)**	−.07 (.703)	.08 (.387)	.03 (.799)	−.03 (.817)	**.01 (.001)**	−.00 (.985)	−**.02 (.005)**	−.28 (.194)
Days of strength training per week	−.10 (.102)	.00 (.870)	−.00 (1.000)	−**.16 (.017)**	−.05 (.496)	.03 (.674)	−.00 (.064)	.03 (.593)	−**.03 (<.001)**	−.07 (.619)
Alcohol consumption										
AUDIT‐C score	−.01 (.965)	.01 (.785)	−.07 (.789)	.15 (.273)	.267 (.092)	−.06 (.716)	**.02 (<.001)**	−.01 (.968)	−.00 (.947)	−.50 (.104)
Drinks (category) consumed on a typical days drinking	−.07 (.158)	−.00 (.703)	.02 (.825)	**.11 (.031)**	.04 (.454)	−.01 (.915)	**.00 (.007)**	−**.23 (<.001)**	.00 (.312)	−.18 (.180)
Days (category) alcohol consumed per month	−.10 (.210)	.01 (.632)	.03 (.823)	.01 (.899)	.12 (.171)	−.02 (.863)	**.01 (<.001)**	**.19 (.012)**	.00 (.884)	−.19 (.278)
Smoking and vaping										
Relapse among baseline smokers (odds ratio, 95% CI)	OR = .84 (.25, 2.84)	OR = .95 (.78, 1.16)	OR = 1.28 (.12, 13.91)	OR = .62 (.18, 2.16)	OR = .19 (.02, 1.57)	OR = .66 (.12, 3.50)	**OR = .94 (.91, 0.97)**	OR = 3.0 (.76, 11.6)	OR = 1.02 (.94, 1.11)	OR = 1.53 (.14, 17.16)
Cigarettes per day (among smokers)	.24 (.852)	−**.40 (.044)**	1.20 (.481)	.28 (.818)	.34 (.843)	−1.74 (.206)	.07 (.157)	1.55 (.229)	.08 (.328)	.71 (.837)
E‐cigarette use frequency (category)	.13 (.793)	.04 (.598)	−^d^	−.61 (.175)	.55 (.327)	.29 (.607)	.03 (.097)	−.06 (.889)	−.01 (.717)	−1.00 (.284)

Note

^a^ The lower the decile, the greater the deprivation.

^b^ All models were adjusted for smoking status as it predicted missing daily surveys.

^c^ Unstandardized regression coefficient unless otherwise stated.

^d^ Zero cell count for e‐cigarette use so covariate excluded.

Bold denotes statistical significant.

Using multiple linear regression, we found that reductions in fruit and vegetables were independently associated with lower age (vegetables unstandardized regression coefficient [B] = .01, *p *< .001; fruit *B* = .02, *p *< .001) and higher body mass index (BMI) at baseline (vegetables *B* = −.01, *p *= .014; fruit *B* = −.01, *p *= .040) (Table 3). White ethnicity was also independently associated with reduced consumption of fruit (*B *= .33, *p *= .027), although only a small proportion (4.3%) of participants were non‐White. Increased consumption of high sugar foods during lockdown was associated with lower age (*B *= −.01, *p *< .001), being female (*B *= .18, *p *= .030) and a higher BMI (*B *= .02, *p *= .003). Finally, reduced self‐rated diet quality during lockdown was associated with lower age (*B *= .01, *p *< .001), being female (*B *= −.21, *p *< .001), and higher BMI (*B *= −.01, *p *= .045).

### Changes in physical activity

There were reductions across the sample in the number of days per week people did 30 minutes or more of self‐reported MVPA (mean difference −.57, 95% CI −0.73, −0.40) but a slight increase in the number of days undertaking strength training (mean difference 0.21, 95% CI 0.09, 0.34) (Table 2 ). Reductions in MVPA appeared to be higher among those with a COVID‐19 at‐risk condition (−.91, 95% CI −1.22, −0.60) and higher deprivation (−.83, 95% CI −1.09, −0.57) relative to the sample as a whole, though similar for those with a mental health issue (−.55, 95% CI −1.18, 0.78). Increases in strength training were observed only among the higher deprivation subgroup. Removing those who completed 3 or fewer daily surveys (Table S1) attenuated the mean difference in MVPA slightly (−0.39) and slightly increased the mean difference in days strength training (0.33) but did not change the overall conclusion. The removal of all but those completing 7 out of 7 daily surveys did not meaningfully change the findings further.

Negative binomial regression models found that reductions in days of MVPA were independently associated with having a COVID‐19 at‐risk condition (*B *= −.19, *p *= .039), greater deprivation (*B *= .03, *p *= .028), being younger (*B *= .01, *p *< .001), and having a higher BMI (*B *= −.02, *p *= .005) (Table 3). Keyworkers (*B *= −.16, *p *= .017) and those of higher BMI (*B *= −.03, *p *< .001) reported greater reductions in strength training post‐COVID‐19.

### Changes in alcohol consumption

The total AUDIT‐C score increased on average for the sample post‐COVID‐19 relative to the pre‐COVID‐19 AUDIT‐C score (mean difference .25, 95% CI 0.13, 0.37) (Table 2). While there was no change found for the typical number of drinks consumed on a typical days drinking category (−.03, 95% CI −0.09, 0.02), there was an increase in the number of days alcohol consumed per month category (.27, 95% CI 0.21, 0.34). The mean change scores were similar among key subgroups. Removing participants with fewer than 15 completed daily surveys for the 30 days used to calculate AUDIT‐C scores led to an increase in the AUDIT‐C score mean difference (0.52) and the days of alcohol consumed category mean difference (0.47) but made little difference to the typical number of drinks mean difference (−.04) (Table S1). No further meaningful changes were observed when removing participants with fewer than 25 completed daily surveys.

Only older age was found to be associated with an increase in AUDIT‐C score (*B *= .02, *p *< .001), using multiple linear regression (Table 3). Being a keyworker (*B *= .11, *p *= .031), older (*B *= .00, *p *= .007), and male (*B* = −.23, *p *< .001) was associated with a greater number of drinks consumed on a typical day’s drinking, although the residuals from the regression model were significantly non‐normal, and transformation of the dependent variable did not improve this. Consuming alcohol on a greater number of days was associated with being older (*B *= .01, *p *< .001) and female (*B *= .19, *p *= .012).

### Changes in smoking and e‐cigarette use

At baseline, 1.0% of all participants reported quitting since the COVID‐19 pandemic started (Table 1). In terms of changes in smoking status since baseline, 6.1% (5/82) of baseline smokers with daily survey data did not report smoking in the first 30 days of daily surveys. Among those who at baseline reported having quit since COVIID‐19, quit before COVID‐19 or being a non‐smoker, 40.0% (4/10), 5.6% (15/267), and 1.8% (11/625) reported smoking in the first 30 days of the daily surveys, respectively (not shown in table).

Across the whole sample, there was no evidence of a difference in smoking post‐COVID‐19 lockdown relative to before the pandemic (1.3%, 95% CI −1.4%, 3.9%) (Table 2). The same was found for the number of cigarettes smoked on average per day among smokers (−0.00, 95% CI −0.96, 0.96) (Table 2). This was similar across key subgroups. Likewise, there was no change in the proportion of participants who used an e‐cigarette either for the whole sample, with 5.3% pre and 4.3% post‐COVID‐19 reporting use (−1.0%, 95% CI −2.8%, 0.9%), or key subgroups. There were no meaningful differences to these findings when removing those who completed fewer than 50% of daily surveys (Table S1) of the more extreme missing data scenario.

Using multiple logistic regression, we found, among baseline ex‐smokers, that younger age (odds ratio = 0.94, 95% CI 0.91, 0.97) was associated with relapse during the first 30 days of daily surveys (Table 3). Among smokers, using linear regression, we found higher deprivation (*B *= −.40, *p *= .044) was associated with a higher number of cigarettes smoked per day. None of the covariates predicted e‐cigarette use frequency.

### Changes in substance use

Across the sample as a whole, there was no evidence of a change in monthly substance use (−1.0%, 95% CI −2.6%, 0.7%) (Table 2), which was similar across subgroups and this did not change meaningfully when removing those who completed fewer than 50% of daily surveys (Table S1). Due to too few data points, the Poisson regression model to examine predictors of a change in substance use frequency would not run.

## Discussion

This study identified changes in dietary, physical activity, and alcohol use behaviours during the early phase of the UK COVID‐19 lockdown relative to pre‐pandemic levels. Most changes across the sample and key subgroups were towards a worsening of health behaviours. However, this was not the case for all observed behaviour; there were no changes in the consumption of high sugar foods and small increases were seen in strength training. While there was no discernible change in smoking and vaping behaviour across the sample, 10% of smokers reported cessation as a result of the pandemic, and less than half of these reported any smoking one month after baseline.

The most notable change was for diet and nutrition behaviour. Fruit and vegetable consumption is consistently associated with survival and mortality, body weight maintenance and the risk of many chronic conditions, and in particular cardiovascular diseases (Angelino et al., 2019; Bellavia, Larsson, Bottai, Wolk, & Orsini, 2013; Bertoia et al., 2015). On average, there was a reduction of almost one portion of fruit and vegetables consumed per day in our cohort and this was highest among younger and higher BMI participants. If sustained, this one portion decrease in fruit and vegetable intake would be associated with a 4–6% increased risk of all‐cause mortality and cardiovascular diseases (Schwingshackl et al., 2017; Wang et al., 2014). The decreased fruit and vegetable intake was reflected in reductions in self‐reported diet quality, which was associated with being female, as well as with younger age and higher BMI, as was the consumption of high sugar foods. However, contrary to the Food Standards Agency UK COVID‐19 consumer tracker report (FSA, 2020), we found no overall change in the consumption of high sugar foods during lockdown relative to pre‐pandemic levels, which may reflect the more granular nature of our question and dietary capture frequency, or the sampled population.

There was a 20% reduction in days with 30 minutes or more moderate to vigorous physical activity (MVPA) observed across the sample but a 15% increase in strength training per week. The change in MVPA may reflect the restrictions on leaving the home imposed early on during lockdown. The finding that those with a COVID‐19 at‐risk condition were less likely to undertake MVPA reinforces this, as many of this group will have been asked to shield and not leave the home for exercise. These findings correspond to other European surveys of heath behaviours during lockdown, also reporting decreased MVPA but increased strength training (Di Renzo et al., 2020), although other work suggests some subgroups increased their activity levels during lockdown (Constandt et al., 2020). Other characteristics associated with a reduction in MVPA included higher BMI and higher deprivation, as found among US adults (Dunton et al., 2020). Together, the evidence indicates that those groups at greatest risk from COVID‐19 reduced their activity the most. This could reduce their immune function (Noz et al., 2019) and increase deconditioning and functional decline, particularly among older people.

For alcohol, the data supported the anecdotal reports of increases in drinking during lockdown relative to pre‐pandemic (Inman, 2020). Our data showed changes in drinking frequency as likely to be driving the increase in overall AUDIT‐C score rather than the number of drinks consumed on a typical day’s drinking. Although on average most participants would not be classified as hazardous drinkers, any increase in the amount of alcohol consumed would increase an individual’s risk of alcohol‐related chronic disease (Mostofsky et al., 2016). Contrary to the other behaviours, being older was associated with an unhealthy change in drinking behaviour. Drinking frequency is associated with older age in high‐income countries including England and Scotland, though quantity consumed is usually higher among younger age groups (Chaiyasong et al., 2018). A more complex picture was found for gender; women drank more frequently but men drank more quantity in lockdown. Drinking in response to COVID‐19‐related psychological distress has been found to be higher for women than men (Biddle, Edwards, Gray, & Sollis, 2020; Rodriguez, Litt, & Stewart, 2020), but whether this explains gender differences in drinking in our cohort requires further analysis.

With fewer participants at baseline who smoked, vaped, or used substances compared to other behaviours, our data are less precise for these. There was little difference in smoking, vaping, and substance use rates between the two periods based on aggregated data, in contrast to other early release findings reporting increased substance use among those who use substances (Crew, 2020). Focusing on individuals longitudinally, we observed some shifting of smoking status. A small proportion of baseline smokers (6%) did not report any smoking in the preceding month, suggesting some or all of them had stopped smoking, in line with reported increases in population level quit attempts in the United Kingdom (ASH, 2020). At the same time, some baseline ex‐smokers reported relapse, as did a small proportion who described themselves at baseline as non‐smokers, supporting the hypothesis that stress can be a causal mechanism for relapse.

### Strengths and limitations

This study began recruiting after the UK lockdown due to the COVID‐19 pandemic and so we did not have real‐time data collected from before the pandemic hit the United Kingdom for comparison purposes. We therefore relied on retrospective recall in order to obtain this data. This resulted in two limitations. Firstly, this could have introduced recall bias. However, we rapidly set the study up after the UK lockdown, which started on 23^rd^ March 2020, and the majority of participants completed the baseline questionnaire on 8^th^ April just over two weeks later, and started their daily surveys on 10^th^ April. Therefore, we were able to minimize the recall period. Furthermore, many validated measures, such as the AUDIT‐C, rely on retrospective recall. We therefore felt this bias was not likely to have had a major influence on the findings. Secondly, comparing values derived from daily EMA with values derived from retrospective recall may have introduced bias. It is reassuring that our findings correspond with national surveys with data collected both pre‐ and post‐pandemic (Niedzwiedz et al., 2020), suggesting also that this did not have a major influence on the findings. Furthermore, this would not likely have affected the predictors analysis which examined relative change between participants during lockdown, while adjusting for pre‐pandemic scores. A further limitation was reliance on self‐reported measurement.

The use of daily surveys to assess health behaviours during lockdown is a strength of the study, increasing the validity of the data primarily through the reduction of recall bias. Furthermore, we assessed and accounted for the impact of missing data to ensure findings were sufficiently robust to changes in missing data assumptions. A further strength was assessing multiple health behaviours. By not focusing on a single behaviour, this may have minimized any ‘mere‐measurement effect’, where actual behaviour is influenced by its assessment, as deliberately changing all of the behaviours assessed simultaneously would be challenging. Assessing how these health behaviours change over a longer time period, by analysing the 3 months of daily surveys we collected, will help identify if any initial mere‐measurement effect was occurring in the early stages of data collection. Future work should also investigate longer term trends and within‐person variability in these health behaviours among the cohort.

### Clinical implications

Our findings indicate that on average, the sample’s health behaviours worsened in the early stages of the UK’s COVID‐19 pandemic measures. On the one hand, it is not surprising that restrictions on movement outside of the house and a greater difficulty in obtaining groceries due to a surge in ‘panic buying’ (Nielson, 2020.) or fear in leaving the house may have led to a less healthy lifestyle for many. On the other hand, if short‐term changes remain as longer term habits, then long‐term health could be compromised as a result. As younger people in general displayed more ‘unhealthy’ changes than older people, the net impact on health outcomes of any long‐term changes in habit would be greater as younger people have more life years ahead of them. This question of maintenance of behaviour change remains an important question to address with the full 3 months of EMA data and the planned longer term follow‐up for this cohort.

Another important finding was that some of the people at greater risk of COVID‐19 demonstrated the most unhealthy behaviour change. Having a higher BMI consistently predicted a worsening of dietary behaviours and a reduction in physical activity, which was also observed for those with a COVID‐19 at‐risk health condition and those living in more deprived neighbourhoods. Targeting at‐risk populations for health behaviour change, particularly those with higher BMI, which formed the focus of a Public Health England campaign in July 2020 (Public Health England, 2020f), is clearly warranted. However, other than for physical activity, our findings indicate little difference in behaviour change among our three key subgroups: those with a COVID‐19 at‐risk condition, those from a more deprived neighbourhood, and those with a self‐reported mental health issue. We also found few differences in behaviour change among keyworkers, those with childcare responsibilities, those living alone, or those from Black and ethnic minority groups.

### Conclusion

After the UK’s COVID‐19 pandemic lockdown and social distancing measures were implemented, we observed moderate reductions in diet quality and moderate to vigorous physical activity, moderate increases in alcohol, small increases in strength training and little change in smoking, vaping and substance use behaviour. Several characteristics were associated with greater unhealthy behaviour change, notably being younger and having a higher BMI, though there were few differences by COVID‐19 at‐risk health condition, deprivation or self‐reported mental health condition. Further investigation into changes in health behaviours over time in this cohort and their impact on physical and mental health will be assessed using the 3 months of daily surveys and the 3‐, 6‐, and 12‐month follow‐ups.

## Conflict of interest

All authors declare no conflict of interest.

## Author contributions

Felix Naughton, (Conceptualization; Data curation; Formal analysis; Funding acquisition; Investigation; Methodology; Project administration; Supervision; Writing – original draft; Writing – review & editing) Emma Ward (Conceptualization; Data curation; Methodology; Project administration; Writing – review & editing) Mizanur Khondoker (Formal analysis; Methodology; Writing – review & editing) Pippa Belderson (Conceptualization; Investigation; Writing – review & editing) Anne Marie Minihane (Conceptualization; Funding acquisition; Investigation; Methodology; Writing – review & editing) Jack Dainty (Data curation; Formal analysis; Writing – review & editing) Sarah Hanson (Conceptualization; Methodology; Writing – review & editing) Richard Holland (Conceptualization; Methodology; Writing – review & editing) Tracey Brown (Conceptualization; Writing – review & editing) Caitlin Notley (Conceptualization; Funding acquisition; Investigation; Methodology; Project administration; Supervision; Writing – original draft; Writing – review & editing).

## Supporting information


**Table S1** Health behaviours pre‐COVID and in the first week of daily surveys during the UK COVID pandemic for all participants and for key subgroups who completed at least 50% of daily surveys used for post‐COVID measure.

## Data Availability

The data that support the findings of this study are available from the corresponding author upon reasonable request.
