# Supplementary material for: Health behaviour change during the UK COVID‐19 lockdown: Findings from the first wave of the C‐19 health behaviour and well‐being daily tracker study
Source: Br J Health Psychol. 2021 Jan 6;26(2):624–43. doi: 10.1111/bjhp.12500 (PMC9291054; doi:10.1111/bjhp.12500)
Supplement: Supplementary file 1 — Table S1 Health behaviours pre‐COVID and in the first week of daily surveys during the UK COVID pandemic for all participants and for key subgroups who completed at least 50% of daily surveys used for post‐COVID measure. [file BJHP-26-624-s001.docx]

Table S1 Health behaviours pre-COVID and in the first week of daily surveys during the UK COVID pandemic for all participants and for key subgroups who completed at least 50% of daily surveys used for post-COVID measure

|  | **C19 at risk health condition** | | | **High deprivation (IMD deciles 1-5)** | | | **Mental Health issue** | | | **All participants** | | |
| --- | --- | --- | --- | --- | --- | --- | --- | --- | --- | --- | --- | --- |
| **Behaviour** | **Pre^a^** | **Post^b^** | **Difference (95% CI)** | **Pre^a^** | **Post^b^** | **Difference (95% CI)** | **Pre^a^** | **Post^b^** | **Difference (95% CI)** | **Pre^a^** | **Post^b^** | **Difference (95% CI)** |
| *Diet and nutrition*^c^ |  |  |  |  |  |  |  |  |  |  |  |  |
| Vegetable portions per day | 3.2 | 2.9 | -0.33  (-0.48, -0.19) | 3.1 | 2.8 | -0.34  (-0.47, -0.21) | 2.6 | 2.5 | -0.07  (-0.36, 0.22) | 3.2 | 2.9 | -0.30  (-0.38, -0.22) |
| Fruit portions per day | 2.5 | 2.0 | -0.54  (-0.69, -0.40) | 2.4 | 1.9 | -0.48  (-0.60, -0.36) | 2.2 | 1.6 | -0.60  (-0.86, -0.32) | 2.4 | 1.9 | -0.56  (-0.64, -0.49) |
| High sugar portions per day | 1.9 | 1.9 | -0.04  (-0.23, 0.15) | 2.2 | 2.1 | -0.07  (-0.23, 0.08) | 2.3 | 2.2 | -0.09  (-0.53, 0.36) | 2.1 | 2.1 | -0.01  (-0.10, 0.08) |
| Self-rated diet quality | 3.2 | 2.7 | -0.42  (-0.53, -0.31) | 3.0 | 2.6 | -0.37  (-0.46, -0.28) | 2.6 | 2.3 | -0.27  (-0.52, -0.01) | 3.2 | 2.7 | -0.47  (-0.52, -0.41) |
| *Physical activity*^c^ |  |  |  |  |  |  |  |  |  |  |  |  |
| Days of ≥30 mins MVPA per week | 3.4 | 2.7 | -0.73  (-1.04, -0.41) | 3.2 | 2.6 | -0.62  (-0.89, -0.36) | 2.5 | 2.0 | -0.48  (-1.13, 0.17) | 3.3 | 2.9 | -0.39  (-0.56, -0.23) |
| Days of strength training per week | 1.4 | 1.5 | 0.19  (-0.06, 0.44) | 1.2 | 1.6 | 0.41  (0.20, 0.62) | 0.8 | 1.3 | 0.44  (-0.03, 0.91) | 1.3 | 1.6 | 0.33  (0.20, 0.46) |
| *Alcohol consumption*^d^ |  |  |  |  |  |  |  |  |  |  |  |  |
| AUDIT-C score | 2.6 | 3.0 | 0.43  (0.21, 0.64) | 2.7 | 3.2 | 0.49  (0.29, 0.69) | 2.1 | 2.5 | 0.38  (-0.06, 0.81) | 2.8 | 3.3 | 0.52  (0.41, 0.64) |
| Drinks (category) consumed on a typical days drinking^e^ | 1.3 | 1.3 | -0.02  (-0.11, 0.64) | 1.4 | 1.4 | -0.03  (-0.12, 0.05) | 1.3 | 1.2 | -0.11  (-0.33, 0.10) | 1.4 | 1.4 | -0.04  (-0.09, 0.02) |
| Days (category) alcohol consumed per month^f^ | 3.1 | 3.5 | 0.41  (0.29, 0.54) | 3.1 | 3.5 | 0.43  (0.33, 0.54) | 2.6 | 3.1 | 0.50  (0.23, 0.77) | 3.1 | 3.6 | 0.47  (0.41, 0.54) |
| *Smoking and vaping*^g^ |  |  |  |  |  |  |  |  |  |  |  |  |
| Reported smoking | 7.5% | 9.1% | 1.7%  (-3.4%, 6.7%) | 9.6% | 12.0% | 2.4%  (-2.7%, 7.5%) | 29.2% | 16.7% | -12.5%  (-28.6%, 4.4%) | 7.6% | 9.7% | 2.1%  (-0.6%, 4.9%) |
| Cigarettes per day (among smokers) | 8.8 | 7.9 | 0.39  (-1.83, 2.62) | 9.6 | 10.6 | 1.17  (-0.57, 2.91) | 11.0 | 10.4 | -0.60  (-3.05, 1.86) | 8.1 | 8.6 | 0.48  (-0.61, 1.57) |
| Any e-cigarette use | 5.0% | 5.4% | 0.4%  (-3.7%, 4.6%) | 5.1% | 5.1% | 0.0%  (-3.7%, 3.7%) | 2.1% | 0.0% | -2.1%  (-10.9%, 5.5%) | 4.6% | 4.0% | -0.6%  (-2.7%, 1.4%) |
| *Substance use*^g^ |  |  |  |  |  |  |  |  |  |  |  |  |
| Any substance use | 2.9% | 3.3% | -0.4%  (-3.0%, 3.9%) | 5.1% | 5.5% | 0.3%  (-3.4%, 4.2%) | 2.1% | 0.0% | -2.1%  (-10.9%, 5.6%) | 3.2% | 3.1% | -0.1%  (-1.9%, 1.6%) |

^a^ Self-reported average portions per day/diet quality before COVID-19 pandemic, collected at baseline (early April 2020)

^b^ Mean self-reported portions consumed per day/diet quality collected daily over the first seven daily surveys after baseline (April 2020)

^c^ Denominators were: all participants (N=901); C-19 Risk (n=262); High deprivation (n=325); Mental health issue (n=54)

^d^ Denominators were: all participants (N=805); subgroups vary for each measure - C-19 Risk (n=225, though some measures higher); High deprivation (n=285, though some measures higher); Mental health issue (n=45, though some measures higher)

^e^ Only includes data from those participants reporting alcohol consumption (‘pre’ [baseline] n=879, ‘post’ n=794). AUDIT-C drinking frequency categories: 1= 1-2 drinks, 2= 3-4 drinks, 3= 5-6 drinks, 4= 7-9 drinks, 5= 10+ drinks

^f^ AUDIT-C days drinking categories: 1=never, 2=once a month, 3= 2-4 times per month, 4= 2-3 times per week, 5= 4+ times per week

^g^ Denominators were: all participants (N=805); C-19 Risk (n=241); High deprivation (n=292); Mental health issue (n=48)
